# *Bombyx mori* nucleopolyhedrovirus (BmNPV) *Bm64* is required for BV production and *per os* infection

**DOI:** 10.1186/s12985-015-0399-9

**Published:** 2015-10-24

**Authors:** Lin Chen, Yunwang Shen, Rui Yang, Xiaofeng Wu, Wenjun Hu, Guoxin Shen

**Affiliations:** Sericultural Research Institute, Zhejiang Academy of Agricultural Sciences, Hangzhou, 310021 China; Lab of Silkworm Biotechnology, College of Animal Sciences, Zhejiang University, Hangzhou, 310058 China

**Keywords:** *Bombyx mori* nucleopolyhedrovirus (BmNPV), *Bm64*, Budded virus production, Occlusion-derived virus formation, *per os* infection

## Abstract

**Background:**

*Bombyx mori* nucleopolyhedrovirus (BmNPV) *orf64* (*Bm64*, a homologue of *ac78*) is a core baculovirus gene. Recently, Li et al. reported that Ac78 was not essential for budded viruses (BVs) production and occlusion-derived viruses (ODVs) formation (Virus Res 191:70–82, 2014). Conversely, Tao et al. demonstrated that Ac78 was localized to the BV and ODV envelopes and was required for BV production and ODV formation (J Virol 87:8441–50, 2013). In this study, the function of *Bm64* was characterized to determine the role of *Bm64* in the BmNPV infection cycle.

**Method:**

The temporal expression of *Bm64* was examined using total RNA extracted from BmNPV-infected BmN cells at different time points by reverse-transcription PCR (RT-PCR) and 5’ RACE analysis. To determine the functions of *Bm64* in viral replication and the viral phenotype throughout the viral life cycle, a deletion virus (vBm^64KO^) was generated via homologous recombination in *Escherichia coli*. Viral replication and BV production were determined by real-time PCR. Electron microscopy was used to detect virion morphogenesis. The subcellular localization of Bm64 was determined by microscopy, and *per os* infectivity was used to determine its role in the baculovirus oral infection cycle.

**Results:**

Viral plaque and titer assay results showed that a few infectious BVs were produced by vBm^64KO^, suggesting that deletion of *Bm64* affected BV production. Viral DNA replication was detected and polyhedra were observed in vBm^64KO^-transfected cells. Microscopy analysis revealed that Bm64 was predominantly localized to the ring zone of the nuclei during the infection cycle. Electron microscopy showed that *Bm64* was not essential for the formation of ODVs or the subsequent occlusion of ODV into polyhedra. The *per os* infectivity results showed that the polyhedra of vBm^64KO^ were unable to infect silkworm larvae.

**Conclusion:**

In conclusion, our results suggest that *Bm64* plays an important role in BV production and *per os* infection, but is not required for viral DNA replication or ODV maturation.

**Electronic supplementary material:**

The online version of this article (doi:10.1186/s12985-015-0399-9) contains supplementary material, which is available to authorized users.

## Background

The family Baculoviridae is composed of insect-specific DNA viruses containing covalently closed, double-stranded DNA genomes ranging from 80 to 180 k bp with 90 to 180 open reading frames (ORFs). This viral family is divided into four genera (*Alphabaculovirus*, *Betabaculovirus*, *Gammabaculovirus* and *Deltabaculovirus*) that include lepidopteran-specific baculoviruses, lepidopteran-specific granuloviruses, hymenopteran-specific baculoviruses, and dipteran-specific baculoviruses, respectively [1]. The viral life cycle presents a biphasic infection process generating progeny with two different phenotypes: budded viruses (BVs), which are produced at the initial stage of the multiplication cycle that are responsible for systemic infection inside the insect host [2, 3] and occlusion-derived viruses (ODVs) produced in the late stage of the cycle that are required for the primary infection that takes place in the midgut epithelium cells of the insect host [4, 5]. Finally, mature ODVs are occluded in a protein matrix to form polyhedra that protect the ODVs from the environment [6].

The *Bombyx mori* nucleopolyhedrovirus (BmNPV) *orf64* (*Bm64*) encodes a gene product 110 amino acids in length [7]. Its homologs are present in all of the sequenced baculovirus genomes and are assigned as a baculovirus core gene [8, 9]. A recent proteomic study determined the protein composition of ODVs of HearNPV and concluded that the homolog of Bm64 was associated with ODVs [10]. Recently, the function of the *Bm64* homologue *Autographa californica* multiple nucleopolyhedrovirus (AcMNPV) *ac78* was analyzed [11, 12]. Tao et al. demonstrated that Ac78 was localized in the BV and ODV envelopes and was required for BV production and ODV formation [12]. A similar phenotype was detected during the investigation of the *Bm64* homologue *Helicoverpa armigera* nucleopolyhedrovirus *ha72*. HA72 was demonstrated to be required for BV production and ODV embedding. Moreover, the IPLKL motif at the N terminus was shown to play an important role in its function [13]. More recently, Li et al. found that Ac78 was not essential for BV production and ODV formation [11], which was a contradictory result. To date, there is no consensus concerning the function of the Bm64 homologue in the viral infection cycle (Additional file 1: Table S1).

Although interruption of *Bm64* resulted in a single-cell infection phenotype [14], the function of *Bm64* in viral infection was not determined in detail. To investigate the role of *Bm64* during BmNPV replication, we generated a *Bm64*-deletion virus (vBm^64KO^) in *Escherichia coli* through homologous recombination. The *Bm64*-deletion decreases BV production but has little effects on viral DNA replication and very late protein expression. Electron micrographs revealed that mature ODVs were detected in the nuclei of vBm^64KO^-transfected cells. *Per os* infection assay results showed that the polyhedra of vBm^64KO^ were unable to infect silkworm 5th instar larvae. Our results suggested that Bm64 played an important role in BV production and *per os* infection but was not required for viral DNA replication or ODV maturation.

## Results

### *Bm64* transcripts in BmN cells after BmNPV infection

**Fig. 1:**
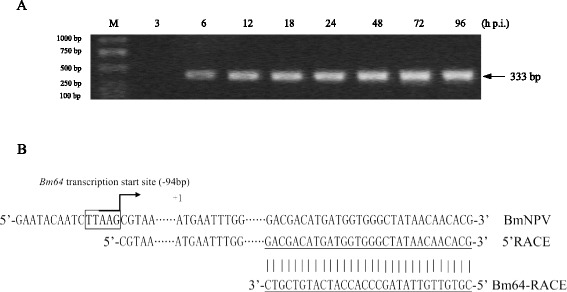
Transcriptional analysis of *Bm64*. **a** RT-PCR analysis of *Bm64* transcripts. Total RNAs were extracted from BmNPV-infected BmN cells at the designated time points. The size of the PCR products is indicated on the right. **b** 5’RACE analysis of the *Bm64* transcriptional start site. The sequence derived from the 5’RACE analysis is shown below. The late promoter, TTAAG (boxed), and the transcriptional start site (arrow) are shown. The translational start codon (ATG) is indicated as +1. The primer Bm64RACE is underlined

For the initial characterization, *Bm64* temporal expression was examined using total RNA extracted from BmNPV-infected BmN cells at different time points by reverse-transcription PCR (RT-PCR) and 5’ RACE analysis. The RT-PCR analyses showed that the 333 bp *Bm64*-specific transcripts were first detected 6 h p.i. (hours post-infection), steadily increased up to 72 h p.i., and remained detectable at 96 h p.i. (Fig. 1a).

A search of the 200 bp 5’ to the predicted start codon of the *Bm64* ORF (ATG) showed the presence of two contiguous late promoter TAAG elements located at positions −12 and −93. 5’ RACE analysis revealed that the *Bm64* mRNA initiated from the G of TTAAG, indicating that the upstream promoter element (−93) was used for *Bm64* transcription (Fig. 1b). The results agreed with the baculovirus transcriptional data [15].

### Construction and analysis of the wild type, *Bm64* knockout and repair BmNPV bacmids

**Fig. 2:**
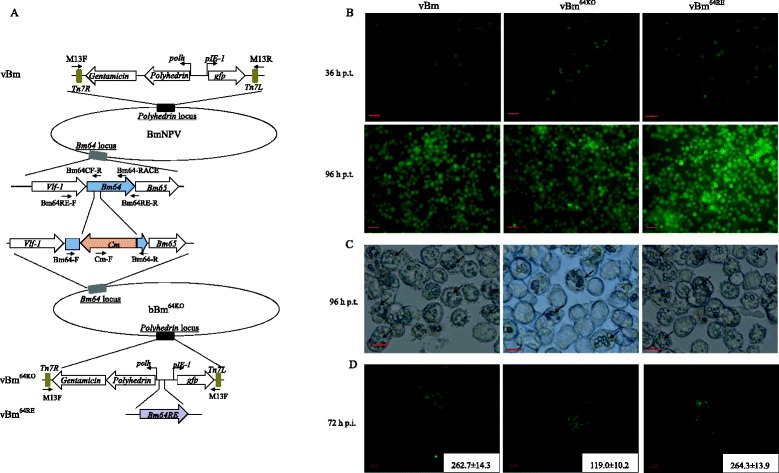
Construction and viral replication analysis of the recombinant bacmids. **a** Schematic diagram of the recombinant bacmids. An 84-bp fragment of the *Bm64* ORF was replaced by a chloramphenicol resistance gene (Cm) via ET homologous recombination in *E. coli* to generate bBm^64KO^. vBm was generated by inserting *polyhedrin* driven by the *polyhedrin* promoter and *gfp* driven by the *ie-1* promoter into the *polh* locus of BmNPV. vBm^64KO^ was constructed by inserting *polyhedrin* and *gfp* into the *polyhedrin* locus of bBm^64KO^ by site-specific transposition. The *Bm64* gene inserted into vBm^64RE^ was driven by its own promoter. **b** Microscopy analysis. Fluorescence microscopy showing the progression of viral infection in BmN cells transfected with vBm, vBm^64KO^ or vBm^64RE^ at 36 and 96 h p.t.. Scale bar, 50 μm. **c** Light microscopy showing the formation of polyhedra in vBm-, vBm^64KO^- or vBm^64RE^ -transfected cells at 96 h p.t. Scale bar, 20 μm. **d** Representative viral plaques from vBm-, vBm^64KO^- and vBm^64RE^-transfected BmN monolayers at 72 h p.t.. The inset shows the means and standard deviations of the plaque sizes from each virus

The *Bm64*-null mutant (bBm^64KO^) was constructed via the λ Red recombination system as previously described [16]. To examine the effect of the *Bm64* deletion on polyhedra morphogenesis and to facilitate the examination of virus infection, the *polyhedrin* and *gfp* genes were transposed into the *polyhedrin* locus of bBm^64KO^ to generate vBm^64KO^ (Fig. 2a). As a positive control, vBm was also generated by inserting *polyhedrin* and *gfp* into the *polyhedrin* locus of the BmNPV bacmid. To confirm that the phenotype resulting from the *Bm64* knockout was not due to genomic effects, we constructed a repair bacmid (vBm^64RE^) containing the *Bm64* ORF driven by its native promoter in addition to the *polyhedrin* and *gfp* sequences.

To determine the effect of the *Bm64* deletion on viral replication, BmN cells were transfected with vBm, vBm^64KO^, or vBm^64RE^. The transfected cells were monitored by fluorescence microscopy. No differences in the numbers of GFP-positive cells were observed among these three samples 36 h p.t. (hours post-transfection), indicating relatively equal transfection levels and efficiencies (Fig. 2b). By 96 h p.t., wide-spread fluorescence was observed in the vBm-, vBm^64KO^-, or vBm^64RE^ -transfected cells.

Light microscopy analysis revealed that polyhedra appeared in all three construct-transfected cells at 96 h p.t. (Fig. 2c), suggesting that viral replication proceeded to very late time points. The bacmid transfection experiments showed that deletion of *Bm64* did not affect OB formation.

Polyhedra were detected in nearly all cells transfected with vBm or vBm^64RE^. However, only 50 % of vBm^64KO^-transfected cells contained polyhedra (Fig. 2c), suggesting that deletion of *Bm64* affected virus infectivity. To monitor the effect of the *Bm64* deletion on viral spread, a GFP fluorescent plaque assay was performed on cell monolayers transfected with vBm, vBm^64KO^, or vBm^64RE^. Only the diameters of well-isolated plaques were measured (16 for each virus) at 72 h p.t.. Both vBm and vBm^64RE^ produced large plaques in the BmN cells, with mean diameters of 262.7 ± 14.32 μm and 264.3 ± 13.94 μm, respectively (Fig. 2d). The vBm^64KO^ plaques were significantly smaller in size, with a mean diameter of 119.0 ± 10.20 μm. This result indicated that deletion of *Bm64* affected virus spread.

### Viral growth curve and viral DNA replication analysis

The viral replication results suggested that the *Bm64* deletion led to a defect in infectious BV production. To confirm these results and to assess the effect of the *Bm64* deletion on virus replication, a viral growth curve analysis was performed using TCID_50_ and qPCR assays. BmN cells were infected with the different constructs, and the BV titers were determined by end-point dilution for TCID_50_ at the selected time points.

**Fig. 3:**
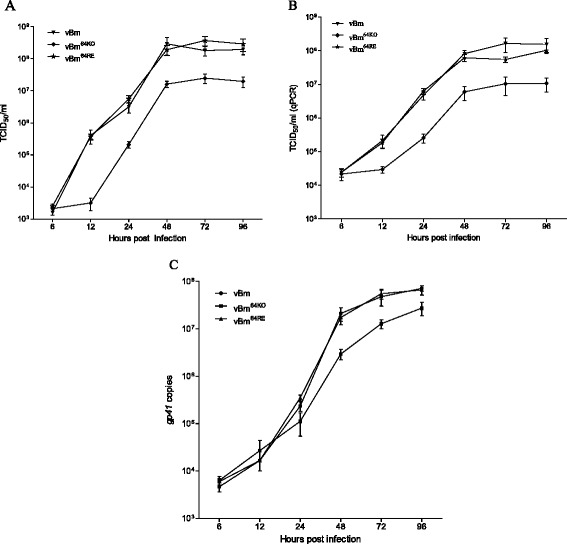
**a** Virus growth curves determined by the TCID_50_ endpoint dilution assays. BmN cells were infected at an MOI of 3 for each virus, and the supernatants were harvested at the selected time points for the titer assay. Each data point was determined from the average of three independent infections; the error bars represent the standard deviations. **b** BV production independent of virion infectivity was determined by quantifying the number of viral genomes by real-time PCR analysis of the supernatants harvested from each bacmid-transfected cell at the designated time points. Each value represents the average of three independent transfections, and the error bars indicate the standard deviations. **c** Real-time PCR analysis of viral DNA replication. BmN cells were transfected with vBm, vBm^64KO^ or vBm^64RE^. At the designated time points, total intracellular DNA was extracted, digested with the restriction enzyme *Dpn* I to eliminate input bacmid DNA, and analyzed by real-time PCR. The graph shows the results of three independent replication assays, with error bars indicating the standard deviations

Newly produced BVs were detectable at 12 h p.i. for vBm or vBm^64RE^. As expected, BmN cells transfected with vBm and vBm^64RE^ revealed a normal increase in virus production (Fig. 3a). However, no infectious BVs were detected up to 12 h p.i. in the vBm^64KO^-infected cells. This result confirmed that Bm64 was required for BV production and viral infection. Furthermore, the *Bm64* repair virus was as sufficient in virus production as the WT virus, confirming that the defect in BV production was not due to genomic effects at the deletion site.

End-point dilution assays were used to determine the production of infectious BVs. Additionally, qPCR analysis was performed to detect BV genomes regardless of infectivity. As expected, a steady increase in BV production was detected up to 96 h p.i. for both vBm and vBm^64RE^. In contrast, an increase in BV production was detected from 48 to 96 h p.i. for vBm^64KO^ -infected cells (Fig. 3b). The qPCR results were in agreement with the TCID_50_ results.

The production of polyhedra (Fig. 2c) suggested that DNA replication occurred in vBm^64KO^ -infected cells [17]. To determine whether *Bm64* had any impact on viral DNA replication, qPCR analysis was performed to compare the levels of viral DNA replication in the vBm-, vBm^64KO^-, and vBm^64RE^-infected cells. Equal amounts of infected BmN cells were collected at the designated time points; then, cell lysates were prepared and total DNA was extracted and subjected to qPCR (Fig. 3c). The results showed that all of the viruses were present at comparable levels up to 24 h p.i.. For vBm and vBm^64R^, the DNA replication levels continued increasing from 24 h p.i. and reached plateaus at 72 h p.i., correlating with the spread of the infection due to the production of BVs. For vBm^64KO^, DNA synthesis increased from 24 h p.i. and by 96 h p.i. the replication level was similar to vBm^64RE^. This result was consistent with the viral replication assays (Fig. 2b).

### Electron microscopy analysis of vBm-, vBm^64KO^-, and vBm^64RE^ -infected cells

**Fig. 4:**
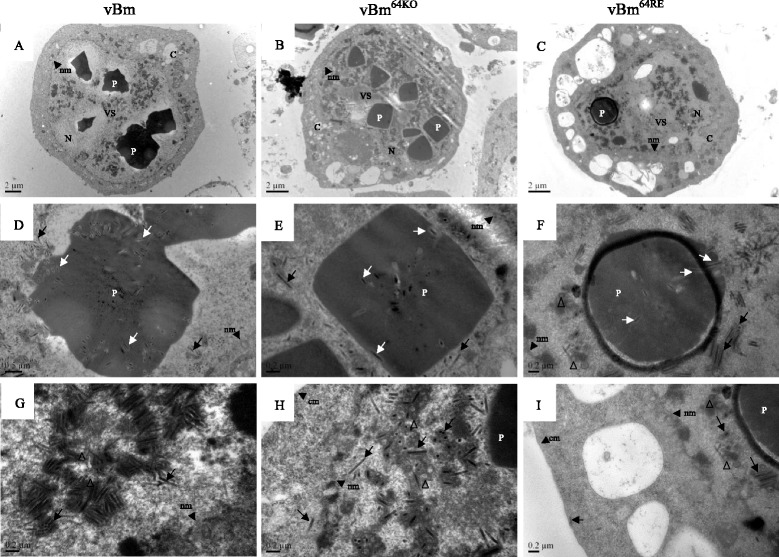
TEM analysis. BmN cells were transfected with vBm (**a**, **d**, and **g**), vBm^64KO^ (**b**, **e**, and **h**), or vBm^64RE^ (**c**, **f**, and **i**), and at 72 h p.i. the cells were analyzed by TEM. Panels **a** **-c** show whole cell low magnification micrographs displaying enlarged nuclei (Nu), virogenic stroma (vs), and polyhedra (P). Panels (**d**, **e**, and **f**) show higher magnification micrographs of polyhedra and the production of ODV (arrows). Panels (**g**, **h**, and **i)** compare nucleocapsids (arrows) and the association of nucleocapsids with virus-induced nuclear microvesicles (triangles). C, cytoplasm; nm, nuclear membrane; cm, cytoplasm membrane

To determine whether the *Bm64* deletion affected virion morphogenesis, electron microscopy analysis was performed with thin sections generated from virus-infected cells at 72 h p.i.. Observations of vBm^64KO^ -infected cells were morphologically indistinguishable from observations of cells transfected with vBm or vBm^64RE^. The vBm^64KO^-infected cells displayed characteristic features of baculovirus infection, such as the VS structure (Fig. 4b), virus-induced nuclear microvesicles (Fig. 4h), the formation of preoccluded virions in the ring zone (Fig. 4e), and mature enveloped ODVs. Polyhedra were also observed in the ring zone of vBm^64KO^-infected cells (Fig. 4b and e). The size and shape of the polyhedra in the vBm^64KO^ -infected cells were similar to those in the vBm^64RE^- or vBm -infected cells (Fig. 4a and c). Enveloped virions were detected in the polyhedra within the ring zone of the vBm^64KO^ -infected cells (Fig. 4e), similar to the vBm^64RE^- or vBm-infected cells (Fig. 4d and f). These observations suggested that Bm64 was not required for the formation of mature ODVs or the subsequent occlusion of ODVs into polyhedra.

### Localization of *Bm64* in BmNPV-infected BmN cells

**Fig. 5:**
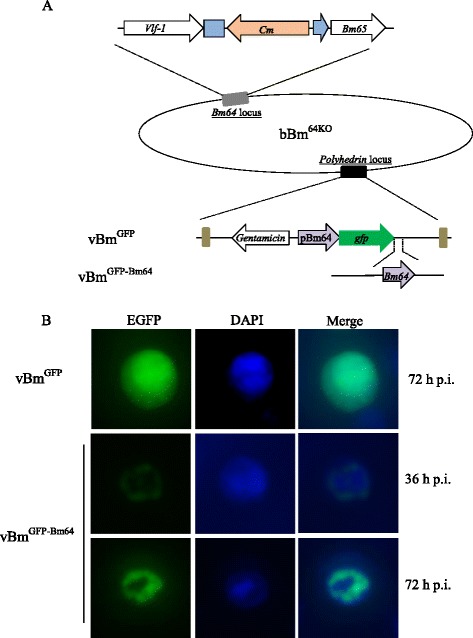
Subcellular localization of the GFP-Bm64 fusion protein in BmN cells. **a** Schematic diagram of the construction of recombinant viruses. A *gfp*-*bm64* chimera under the control of the *polyhedrin* promoter was inserted into the *polh* locus of the vBm^64KO^ bacmid to generate vBm^GFP-Bm64^. The control virus vBm^GFP^ was constructed by transposing *gfp* under control of the *polyhedrin* promoter into the vBm^64KO^ bacmid. **b** Microscopy images of BmN cells infected with vBm^GFP-Bm64^ and vBm^GFP^. BmN cells were infected with vBm^GFP-Bm64^ or vBm^GFP^ at an MOI of 1 and were observed for fluorescence by laser scanning microscopy at 36 or 72 h p.i

Two viruses (vBm^GFP-Bm64^ and vBm^GFP^) were constructed to monitor the subcellular localization of Bm64 (Fig. 5a). In vBm^GFP-Bm64^, Bm64 was expressed in-frame with GFP to produce a GFP-Bm64 fusion protein under the control of the *Bm64* promoter. As a control, GFP alone was expressed under the control of *Bm64* promoter in vBm^GFP^ (Fig. 5a). Fluorescence was detected throughout the vBm^GFP^-infected cells (Fig. 5b). However, the fluorescence was restricted along the inner periphery (ring zone) of the nucleus in the BmN cells infected with vBm^GFP-Bm64^ (Fig. 5b).

### Bm64 is required for *per os* infection

**Fig. 6:**
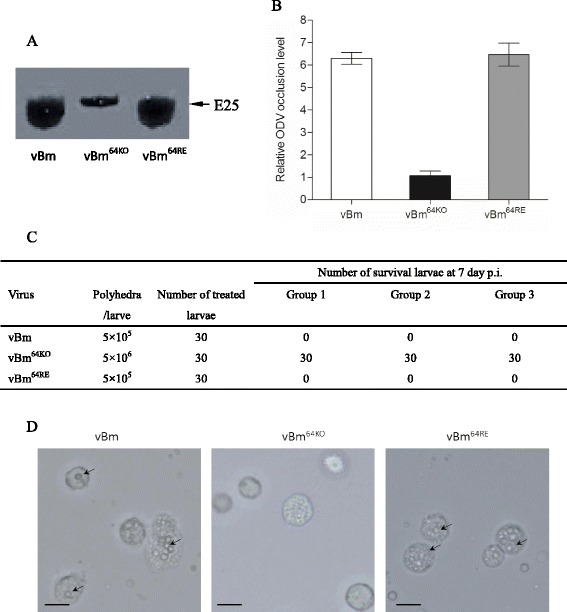
ODV occlusion and *per os* infection assays. **a** Total ODVs were collected from polyhedra and detected by Western blotting with the E25 antibody. **b** The ODV occlusion levels of the three viruses were normalized to vBm^64KO^. **c** Survival analysis of polyhedra of vBm (5 × 10^5^ polyhedra/larva), vBm^64KO^ (5 × 10^6^ polyhedra/larva) or vBm^64RE^ (5 × 10^5^ polyhedra/larva)-infected silkworm larvae. **d** Microscopy analysis of the blood cells of vBm-, vBm^64KO^- or vBm^64RE^-infected silkworm larvae. At 4 days post *per os* infection, the blood cells of infected silkworms were collected and examined by microscopy (Nikon). Polyhedra were indicated by arrows. Scale bar: 10 μm

To investigate whether the *Bm64* deletion had any effect on ODV embedding, ODVs were collected from equal amounts of polyhedra (1.0 × 10^8^) prepared from vBm-, vBm^64KO^-, or vBm^64RE^-transfected BmN cells. The E25 ODV envelope protein was used to detect the ODVs. The result showed that increased amounts of E25 were detected from the vBm and vBm^64RE^ polyhedra compared to the vBm^64KO^ polyhedra, suggesting that the deletion of *Bm64* affected the ODV occlusion efficiency into polyhedra (Fig. 6a).

To examine the effect of the *Bm64* deletion on the infectivity of the ODVs, the prepared polyhedra were administered to newly molted 5th instar silkworm larvae. Dosages of 5.0 × 10^5^ vBm and vBm^64RE^ polyhedra/larva resulted in a 100 % mortality rate. Because the ODV occlusion level of vBm and vBm^64RE^ was approximately 7-fold higher compared to vBm^64KO^ (Fig. 6b), the dosage for vBm^64KO^ was 5.0 × 10^6^ polyhedra/larva. However, ingestion of the polyhedra from vBm^64KO^ did not result in death (Fig. 6c). The blood examination results showed that polyhedra were detected in both the vBm- and vBm^64RE^-infected larvae. In contrast, no polyhedra were detected in the blood cells of the vBm^64KO^-infected larvae (Fig. 6d). These results indicated that Bm64 was required for BmNPV oral infection.

## Discussion

All baculoviruses sequenced to date contained homologues of 37 core genes, suggesting that these genes performed key functions in the baculovirus life cycle [8, 18, 19]. In this study, we investigated the role of a recently identified core gene (BmNPV *Bm64*). We found that *Bm64* played important roles in BV production and *per os* infection but was not required for viral genome replication or mature ODV formation.

Homologues of *Bm64* are found in all baculoviruses, suggesting that this protein is required for a function utilized by all members of the *Baculoviridae* [20]. A Bm64 homologue was detected in the envelope of both ODVs and BVs [10]. At least 5 other proteins in addition to Bm64 are specifically localized to the envelopes of BVs and ODVs, including Ubiquitin, Ac68, E25, PIF-4, and E18 [10]. BV production and ODV formation are not affected by the deletion Ac68 and PIF-4 [21, 22]. In contrast, a *ubiquitin* mutant virus caused a 5-10-fold reduction in BV production, and E25 and E18 were required for efficient BV production and ODV formation [16, 23].

The role of *Bm64* in the context of BmNPV infection in BmN cells was analyzed using the *Bm64* knockout bacmid. End-point dilution and qPCR assays demonstrated that vBm^64KO^ had a defect in BV production. Electron microscopy showed that nucleocapsids produced by vBm^64KO^ were morphologically indistinguishable from those observed for either vBm or vBm^64RE^ (Fig. 4), and mature enveloped ODVs were found in vBm^64KO^ -transfected cells. These results indicated that Bm64 played an important role in BV production but was not required for the formation of mature ODVs.

After nucleocapsids replicate in the nuclei of infected cells, they need to exit in order to spread the infection. They have been suggested to rapidly egress from the nucleus to the cytoplasm and obtain the envelope from the cytoplasmic membrane [18]. Many viral proteins were shown to be essential for this process. Some were required for the egress of the nucleocapsids from the nucleus (e.g., Ac66 [24] and Ac88 [25]). Others that were not required for nucleocapsid egress from the cells affected the viral titer (e.g., Ac109 [26] and Ac34 [27]). Finally, some genes were involved in the transfer of the nucleocapsids to the cytoplasm (e.g., P78/83 [28]). During the baculovirus infection cycle, nucleocapsids undergo intracellular motility driven by actin polymerization; the motility requires at least the viral P78/83 protein and the host Arp2/3 complex [28].

The subcellular localization of Bm64 demonstrated that this protein was primarily distributed in the ring zone of infected nuclei during viral infection (Fig. 5). A recent study demonstrated that *ac78* was required for nucleocapsid egress from the nucleus [12]. Many proteins are localized to the ring zone of infected nuclei, such as Ac76 [29], P33 [13], Ac93 [19], and E25 [30]. The ring zone is very important for nucleocapsid envelopment and egress from the nucleus. Ac78 was demonstrated to interact with P33 in the ring zone; both Ac78 and P33 are BV envelope components, suggesting that BVs obtain these ring zone-localized proteins from the nucleus [13]. The ODV envelope proteins P74, PIF-1, PIF-2, and PIF-3 form a complex on the ODV envelope [31] and are not required for ODV formation and ODV embedding into polyhedra [32], indicating that these proteins are nonessential for the recognition between nucleocapsids and intranuclear microvesicles or between ODVs and polyhedra. Consistent with the previous study, *per os* infectivity assays demonstrated that Bm64 was required for the BmNPV oral infection process, indicating that Bm64 played an important role in ODV primary infection [11].

Our results agree with the findings of Li et al. that *ac78* (*Bm64* homolog) plays an important role in BV production efficient ODV occlusion [11]. However, deletion of *ac78* resulted in a more severe defect for AcMNPV BV infection. A comparison of the predicted amino acid sequences of Bm64 homologues showed that the conservation was very low (Additional file 2: Figure S1) [13], suggesting that the functions of Bm64 homologues during viral infection might differ. The characteristics of baculovirus core genes were demonstrated to be conserved, but they might have different functions in the viral infection cycles.

## Conclusion

In conclusion, this study demonstrates that deletion of Bm64 affects BV production and ODV infectivity but does not affect ODV formation. Although the exact function of Bm64 in nucleocapsid egress from the nucleus and ODV formation is still unclear, our study has provided valuable insight into the baculovirus life cycle.

## Methods

### Bacmid, virus, and cells

The *E. coli* strains BW25113 containing the plasmid pKD46 and BW25141 harboring the plasmid pKD3 (encoding the chloramphenicol resistance gene) were kindly provided by Mary Berlyn (Yale university). The *E. coli* strain DH10H (containing a helper plasmid pMON7124) and DH10BmBac (containing a BmNPV bacmid and a helper plasmid pMON7124) were constructed previously in our lab [33]. BmN cells were cultured at 27 °C in TC-100 insect medium supplemented with 10 % fetal calf serum (Gibco, USA).

### Total RNA preparation, RT-PCR and 5’ rapid amplification of cDNA ends (5’RACE) analysis

BmN cells were infected with BmNPV at a multiplicity of infection (MOI) of 5 50 % tissue culture infective doses (TCID_50_). At various time points post-infection (p.i.), total cellular RNA was isolated according to the manufacturer’s instructions (RNeasy mini kit, Qiagen, Germany). Reverse transcription-PCR (RT-PCR) was performed with an EasyScript First-Strand cDNA Synthesis SuperMix kit (Transgen, China) using 2.0 μg of total RNA as the template for each time point. Synthesis of first-strand DNA complementary to the mRNA (cDNA) was performed using the avian myeloblastosis virus reverse transcriptase and oligo(dT) primers according to the manufacturer’s instructions. The Bm64-specific primers Bm64-F (5’-ATGAATTTGGACGTGCCATAC-3’) and Bm64-R (5’-CTCGATTAACCACAATGAACGTCTAGAGC-3’) were used for PCR amplification to detect the *Bm64* transcripts.

To characterize *Bm64*, its temporal expression was examined by 5’RACE analysis. The 5’RACE procedure was performed using the Smarter^TM^ RACE cDNA Amplification Kit (Clontech, USA) with 1 μg of purified total RNA isolated from BmNPV-infected cells at 48 h p.i.. A Bm64-specific primer (Bm64-RACE, 5’-GCTTGCTCCTGTTTGAGTTCAG-3’) was used for cDNA synthesis and PCR amplification following the manufacturer’s instructions. The PCR products were gel purified and cloned into the pGEMT-easy vector (Promega, Madison, USA).

### Generation of the *Bm64*-knockout BmNPV bacmid

A *Bm64*-knockout BmNPV bacmid was generated as previously described [16]. A chloramphenicol resistance gene (*Cm*) was amplified using Bm64KO-F (5’- GACACGTTGCTCGTCGTGTTGTTATAGCCCACCATCATGTCGTCTATTGGGTGTAGGCTGGAGCTGCT-3’) and Bm64KO-R (5’- ACATGAATTTGGACGTGCCATACTATCGGTTGGGCAACCACGAAAAGCATATGAATATCCTCCTTAG -3’) with pKD3 as the template. These primers contained 50 and 47 bp sequences homologous to the upstream and downstream flanking regions (underlined sequences) of *Bm64*, respectively; a stop codon (black box) was also introduced. The *Cm* cassette PCR fragment was gel purified using a QIAquick PCR purification kit (Qiagen, USA) and electroporated into *E. coli* BW25113 cells containing the BmNPV bacmid. The electroporated cells were incubated at 37 °C for 3 h in 1 ml of SOC medium (2 % Bacto tryptone, 0.5 % Bacto yeast extract, 10 mM NaCl, 2.5 mM KCl, 10 mM MgCl_2_, 10 mM MgSO_4_, and 20 mM glucose) and plated onto agar medium containing 7 μg/ml chloramphenicol and 50 μg/ml kanamycin. The plates were incubated at 37 °C overnight. Colonies resistant to both chloramphenicol and kanamycin were selected and confirmed with the primers Bm64-R (5’- CTCGATTAACCACAATGAACGTCTAGAGC -3’) and Cm-F (5’-TTGTTACACCGTTTTCCATGAGC-3’) to detect the correct insertion of the *Cm* in the region of the *Bm64* locus.

The recombinant bacmids confirmed by PCR and sequencing were selected and designated bBm^64KO^. The identified bBm^64KO^ was extracted and electro-transformed into *E. coli* DH10βH to generate DH10Bm^64KO^ cells containing both the *Bm64*-deleted bacmid and the helper plasmid.

### Construction of the *Bm64* knockout, repair, and positive control BmNPV bacmids

The *Bm64* knockout, the repair and the positive control BmNPV bacmids containing *polyhedrin* and *gfp* (enhanced green fluorescence protein gene) were constructed by site-specific transposition as previously described [34]. The pFast-PH-GFP (containing *polyhedrin* and *gfp*) was constructed as described and transformed into electrocompetent DH10BmBac or DH10Bm^64KO^ cells to generate the *Bm64* knockout bacmid (vBm^64KO^) or the positive control bacmid (vBm), respectively.

To construct a repair bacmid (vBm^64RE^), a 531 bp fragment containing the Bm64 gene with its native promoter was amplified using the primers Bm64RE-F (5’-GAAGGCCTCAAGTGTTTGCGCAACGCAAC-3’) and Bm64RE-R (5’-GCTCTAGACGTTCATTGTGGTTAATCGAG-3’). The repair fragments were cloned into the pFast-PH-GFP plasmid to generate pFast-PH-Bm64RE-GFP. pFast-PH-Bm64RE-GFP was used to transpose the parental knockout bacmids to generate the *Bm64* repair bacmid (vBm^64RE^). To confirm vBm^64RE^ by PCR, we used the primer Bm64CF-R (5’-GTTCGCTGGTGATATCATCGTTGAG -3’) located in the deletion sequence of vBm^64KO^. vBm^64RE^ was confirmed with Bm64RE-F/Bm64CF-R. Bacmid DNA was extracted and quantified as described previously [35].

### Viral growth curve analysis and plaque assay

BmN cells (1.0 × 10^6^) were transfected with 1.0 μg of each bacmid (vBm, vBm^64KO^, or vBm^64RE^). At 36 and 96 h p.t., the progression of viral infection was monitored by fluorescence microscopy. A viral plaque assay was performed as previously described [36]. Briefly, BmN cells were plated at a density of 1 × 10^6^ cells/35-mm-diameter well of a six-well plate. The cells were transfected with 10 ng of vBm, vBm^64KO^, or vBm^64RE^ bacmid DNA. Then, the monolayers were overlaid with 1 % low-melting-point agarose for cell culture (Gibco, USA) in complete Grace’s medium. The plaques were photographed and measured 72 h p.t..

### Analysis of the viral growth curve

To evaluate the viral replication of vBm, vBm^64KO^, and vBm^64RE^, BmN cells were infected in triplicate with each virus (vBm, vBm^64KO^, or vBm^64RE^) at an MOI of 3. After 1 h of incubation, the cells were washed twice and the medium was replaced with fresh TC100 medium. Supernatants were collected at the indicated time points (6, 12, 24, 48, 72, and 96 h p.i.), and the titers were determined by an end point dilution assay on BmN cells.

TCID_50_ was used to determine the infectious virions, whereas quantitative real-time PCR (qPCR) was performed to confirm the baculovirus stocks as previously described [37]. Briefly, an aliquot of each supernatant (250 μl) was processed using a viral DNA kit (Omega, USA). A 2.0 μl aliquot of each purified DNA sample was mixed with 10 μl of SYBR® Premix ExTaq (TaKaRa, Japan) and the qPCR primers in a 20 μl reaction volume. The PCR was performed using the 7300 Real-Time PCR system (ABI, USA) under the following conditions: 95 °C for 30 s and 45 cycles of 95 °C for 5 s and 60 °C for 31 s.

### Quantitative real-time PCR (qPCR) DNA replication assay

To detect viral DNA replication, a qPCR assay was performed as previously described [38]. BmN cells were infected with vBm, vBm^64KO^, or vBm^64RE^ at an MOI of 1 and harvested at different time points. Total DNA was extracted with the Classic Genomic DNA Isolation Kit (Sangon, Canada). Q-PCR was performed with a 500 nM concentration of each primer using the 7300 Real-Time PCR system (ABI, USA) under the following conditions: 95 °C for 30 s and 45 cycles of 95 °C for 5 s and 60 °C for 31 s.

### Transmission electron microscopy (TEM)

BmN cells (5 × 10^6^ cells) were infected with vBm, vBm^64KO^, or vBm^64RE^ at an MOI of 5. At the indicated time point post-infection, the cells were collected and centrifuged at 5000 rpm for 5 min. Then, the cells were fixed, dehydrated, embedded, sectioned, and stained as previously described [16]. The samples were visualized with a TEM Model JEM-1230 at an accelerating voltage of 120 kV.

### Construction of GFP fusion recombinant bacmids and microscopy determination

To monitor the localization of Bm64 in BmNPV-infected BmN cells, GFP was fused at the N-terminus of Bm64 under the control of the *Bm64* promoter (pBm64) to create a GFP-Bm64 fusion protein. A recombinant fusion bacmid (vBm^GFP-Bm64^) and a control bacmid (vBm^GFP^) were constructed as previously described [30]. The *Bm64* promoter was PCR-amplified using the primers Bm64pro-F (5’-GACCATGGCAAGTGTTTGCGCAACGCAAC-3’) and Bm64pro-R (5’-CGGAATTCCCACGTCCAAATTCATGTTTACAAC-3’). The enhanced green fluorescent protein (*egfp*) was amplified with the primers EGFP-F (5’-AAGCTTCGCCACCATGGTGAGCAAG-3’) and EGFP-R (5’-GGTACCCTTGTACAGCTCGTCCATG-3’), while *Bm64* was amplified with Bm64-F (5’-GGTACCATGAATTTGGACGTGCCATAC-3’) and Bm64-R (5’- AAGCTTCGTTCATTGTGGTTAATCGAG -3’). BmN cells (1 × 10^6^) were transfected with 1 μg of vBm^GFP-Bm64^ or vBm^GFP^ DNA. At 96 h p.t., the supernatants were collected, and the BV titers were determined by an endpoint dilution assay. For microscopy analysis, BmN cells (5 × 10^5^) were infected with vBm^GFP-Bm64^ or vBm^GFP^ at an MOI of 1. At 36 and 72 h p.i., the cells were examined with a microscope to analyze the GFP fluorescence.

### Purification of ODVs for western blot analysis

The polyhedra were prepared from the infected cells as previously described [31]. Polyhedra were suspended in DAS buffer (0.1 M Na_2_CO_3_, 166 M NaCl, and 10 mM EDTA, pH 10.5), and the solution was neutralized with 0.5 M Tris–HCl (pH 7.5). After removing the insoluble debris, the ODVs were collected by centrifugation at 50,000 × g for 60 min at 4 °C and resuspended in 0.1× TE at 4 °C.

Protein samples were separated by SDS-polyacrylamide gel electrophoresis (PAGE) with a 12 % acrylamide separating gel. For Western blot analysis, the gels were electroblotted onto nitrocellulose (NC) membranes. Proteins on the membranes were blocked in 25 mM Tris-base (pH 7.4) with 140 mM NaCl, 2.7 mM KCl, 0.05 % Tween-20 (TBS-T) and 5 % milk. For immune detection, the membranes were incubated for 2 h at room temperature with the primary anti-E25 rabbit polyclonal antibody (1:1000). The secondary antibody was added, and the blots were incubated for 2 h prior to three washes in TBS-T. The secondary goat anti-rabbit IgG antibody conjugated with horseradish peroxidase (Amersham Biosciences, Germany) was diluted 1:2000 in TBS-T with 5 % milk. Blots were detected using an enhanced chemiluminescence system (ECL; Thermo, USA) according to the manufacturer’s instructions and analyzed with Image J (http://rsb.info.nih.gov/ij).

### In vivo infectivity assays

The infectivity of the ODVs in vivo was examined by orally inoculating newly molted 5th instar *Bombyx mori* larvae with the polyhedra of vBm, vBm^64KO^, or vBm^64RE^. The polyhedra were purified from transfected BmN cells, and the oral infectivity bioassays were performed as previously described [22]. A cohort of 30 larvae was used for each treatment, and the treatment was repeated in triplicate. Infected larvae were reared with fresh mulberry until all larvae pupated or died. At 4 days post-molt, the blood of the *Bombyx mori* larvae was collected and observed under a microscope to detect the virus infection.

## Additional files

Additional file 1: Table S1. Comparison the roles of Bm64 homologs. (DOCX 16 kb) Additional file 2: Figure S1. Amino acid sequence aligment of 12 Bm64 homologs. (DOCX 253 kb)
