# Supplementary material for: Bombyx mori nucleopolyhedrovirus (BmNPV) Bm64 is required for BV production and per os infection
Source: Virol J. 2015 Oct 24;12:173. doi: 10.1186/s12985-015-0399-9 (PMC4619395; doi:10.1186/s12985-015-0399-9)
Supplement: Additional file 1: Table S1. — Comparison the roles of Bm64 homologs. (DOCX 16 kb) [file 12985_2015_399_MOESM1_ESM.docx]

**Additional file 1: Table S1**

**Table S1:** Comparison the roles of Bm64 homologs.

| Gene name | Virus name | BV production | BV infectivity | ODV occlusion | Publication date |
| --- | --- | --- | --- | --- | --- |
| *Bm64* | BmNPV | Not clear | non-detectable | Not clear | 2010 |
| *ac78* | AcMNPV | non-detectable | non-detectable | non-detectable | 2013 |
| *ha72* | HearNPV | non-detectable | non-detectable | non-detectable | 2014 |
| *ac78* | AcMNPV | detectable | detectable | detectable | 2014 |
