# Supplementary material for: Bombyx mori nucleopolyhedrovirus (BmNPV) Bm64 is required for BV production and per os infection
Source: Virol J. 2015 Oct 24;12:173. doi: 10.1186/s12985-015-0399-9 (PMC4619395; doi:10.1186/s12985-015-0399-9)

**Additional file 2: Figure S1.**

**Figure S1**. Amino acid sequence alignment of 12 Bm64 homologs. The location of the IPLKL motif and a putative FRF were indicated. Ten representative sequences were selected and aligned using CLUSTAL X 1.83 and edited with GeneDoc software. Black shading denotes 100% conservation. Dark gray and light gray shading represents 80 and 60% conservation, respectively.

Supplemented data Figure 1

FRF motif

IPLKL motif


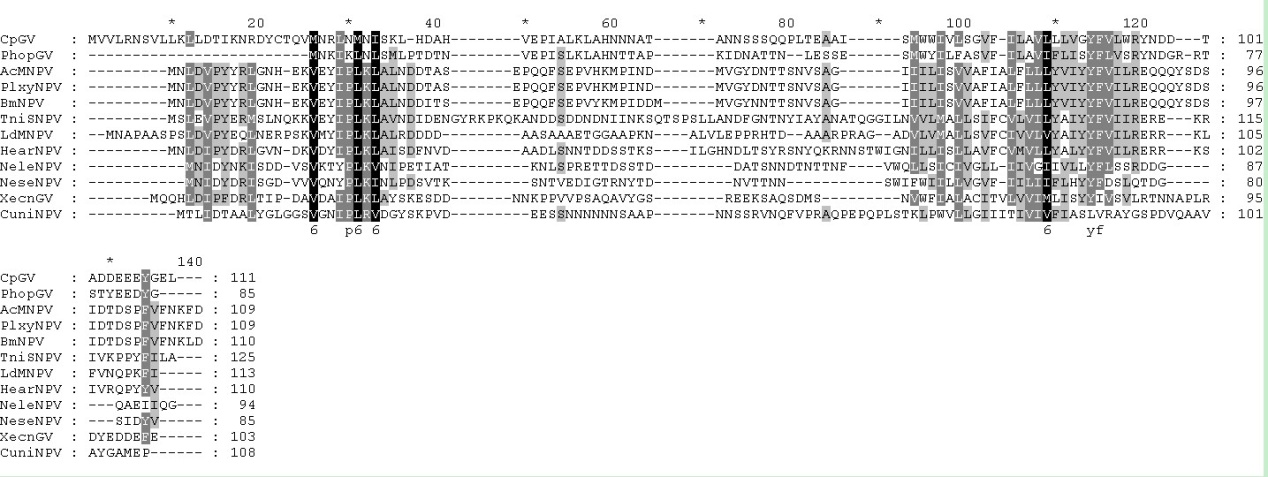

Supplement: Additional file 2: Figure S1. — Amino acid sequence aligment of 12 Bm64 homologs. (DOCX 253 kb) [file 12985_2015_399_MOESM2_ESM.docx]
